# TcZC3HTTP, a regulatory element that contributes to *Trypanosoma cruzi* cell proliferation

**DOI:** 10.1128/spectrum.02880-23

**Published:** 2024-01-25

**Authors:** Bruno Accioly Alves Romagnoli, Aline Castro Rodrigues Lucena, Eden Ribeiro Freire, Isadora Filipaki Munhoz da Rocha, Lysangela Ronalte. Alves, Samuel Goldenberg

**Affiliations:** 1 Gene Expression Regulation Laboratory, Carlos Chagas Institute, Fiocruz PR, Curitiba, Paraná, Brazil; 2 Laboratory for Applied Science and Technology in Health, Carlos Chagas Institute, Fiocruz PR, Curitiba, Paraná, Brazil; 3 Research Center in Infectious Diseases, Division of Infectious Disease and Immunity, CHU de Quebec Research Center, University Laval, Quebec, Canada; George Washington University, Washington, DC, USA

**Keywords:** *Trypanosoma cruzi*, gene expression regulation, cell proliferation, RNA-binding proteins, nutritional stress

## Abstract

Post-transcriptional regulation of gene expression is a critical process for adapting to and surviving *Trypanosoma cruzi*, a parasite with a complex life cycle. RNA-binding proteins (RBPs) are key players in this regulation, forming ribonucleoprotein complexes (messenger ribonucleoproteins) and RNA granules that control transcript stability, localization, degradation, and translation modulation. Understanding the specific roles of individual RBPs is crucial for unraveling the details of this regulatory network. In this study, we generated null mutants of the TcZC3HTTP gene, a specific RBP in the *Trypanosoma* family characterized by a C3H zinc finger and a DNAJ domain associated with RNA and protein binding, respectively. Through cell growth assays, we demonstrated that the absence of TcZC3HTTP or the expression of an additional tagged version impacted epimastigote growth, indicating its contribution to cell proliferation. TcZC3HTTP was found to associate with mRNAs involved in cell cycle and division in epimastigotes, while in nutritionally stressed parasites it exhibited associations with mRNAs coding for other RBPs and rRNA. Furthermore, our analysis identified that TcZC3HTTP protein partners were different during normal growth conditions compared to starvation conditions, with the latter showing enrichment of ribosomal proteins and other RBPs. Therefore, this study provides insights into TcZC3HTTP’s role in the post-transcriptional regulation of gene expression during normal growth and nutritional stress in *T. cruzi*, uncovering its versatile functions in different cellular contexts.

**IMPORTANCE:**

Understanding how *Trypanosoma cruzi*, the causative agent of Chagas disease, regulates gene expression is crucial for developing targeted interventions. In this study, we investigated the role of TcZC3HTTP, an RNA-binding protein, in post-transcriptional regulation. Our findings demonstrate that TcZC3HTTP is relevant for the growth and proliferation of epimastigotes, a stage of the parasite’s life cycle. We identified its associations with specific mRNAs involved in cell cycle and division and its interactions with enzymes and other RNA-binding proteins (RBPs) under normal and starvation conditions. These insights shed light on the regulatory network underlying gene expression in *T. cruzi* and reveal the multifaceted functions of RBPs in this parasite. Such knowledge enhances our understanding of the parasite’s biology and opens avenues for developing novel therapeutic strategies targeting post-transcriptional gene regulation in *T. cruzi*.

## INTRODUCTION

Over several decades, extensive research has established that *Trypanosoma cruzi* and other trypanosomatids primarily rely on post-transcriptional mechanisms to regulate gene expression (1–4). These mechanisms encompass various events related to RNA metabolism, including RNA maturation, nuclear exporting, subcellular localization, translation, and degradation/storage (5, 6).

Messenger RNAs (mRNAs) undergo individual or coordinated regulation within the cell through dynamic interactions with RNA-binding proteins (RBPs). These interactions lead to the formation of ribonucleoprotein complexes (RNPs). Furthermore, RNPs can interact with and exchange components between them, giving rise to larger regulatory complexes known as RNA granules (7). Interactions involving RBPs and their target RNAs are highly dynamic and influenced by environmental changes such as temperature, nutrient availability, and oxidative stress. *T. cruzi* encounters these challenges during its transition within and between mammalian and triatomine hosts (8). The parasite must undergo a coordinated and rapid cellular and molecular response to maintain homeostasis and ensure survival, which includes rearrangements and modulation of RNP complexes. These rearrangements directly impact gene expression. Since RBPs are involved in every step of RNA metabolism, they play a pivotal role as crucial regulators of gene expression (9, 10).

Besides coordinating the cellular RNA levels, many studies in Trypanosomatids that managed to silence, knockout, or overexpress RBPs reported alterations in cellular proliferation, division, and differentiation, indicating that these regulatory proteins also impact major cellular processes (11–18). In *Trypanosoma brucei*, for example, the RBP TbPUF9 silencing decreased bloodstream-form cell growth and increased the number of cells accumulated into the G2/M phase (11). For *T. cruzi*, TcZC3H31 knockout abolished the differentiation into metacyclic trypomastigotes (16), whereas TcZC3H12 disruption presented a higher metacyclogenesis rate and a decrease in cellular proliferation (19). Interestingly, the RBP TcUBP1 overexpression notably amplifies the infectivity rates of *T. cruzi*, primarily through the upregulation and subcellular redistribution of its target transcripts, which mainly encode surface proteins (18).

The bacterial adaptive immune system CRISPR/Cas9 discovery and its use as a genome engineering tool emerged as a promising alternative to perform gene disruption in several organisms, including *T. cruzi* (20–22). Recently, our group developed an approach that allowed us to confirm the first knockout achieved with CRISPR/Cas9 of an RBP in *T. cruzi*, the zinc finger protein TcZC3HTTP, thus unveiling a new perspective to advance in RBP characterization in this organism (23).

Here, we report our findings regarding the disruption of TcZC3HTTP in *T. cruzi*, an RBP exclusive to the *Trypanosoma* family that presents two characteristic domains, a zinc finger C3H and a DNAJ domain related to RNA and protein interaction, respectively. Although about one-third of *T. cruzi*’s C3H zinc finger proteins have more than one domain; this combination that TcZC3HTTP presents seems to be unique within this group (24). In plant species, there are DNAJ-like zinc finger domain proteins involved in the biogenesis and/or maintenance of plastids, but their motifs are not C3H; instead, they are C4 (25).

Modulating TcZC3HTTP expression, we identify that this RBP is related to *T. cruzi* cell proliferation. Next, we evaluated the mRNA targets and protein partners of TcZC3HTTP in normal and nutritional stress conditions. For this purpose, we performed immunoprecipitation assays followed by RNA-seq and mass spectrometry using a protein-tagged version (TcZC3HTTP-3×FLAG). In addition to investigate the effects on the TcZC3HTTP mRNA target and protein partner candidates in the *tczc3http* null mutant population, we assessed total RNA and protein in these conditions.

## RESULTS

### TcZC3HTTP phylogeny

**Fig 1:**
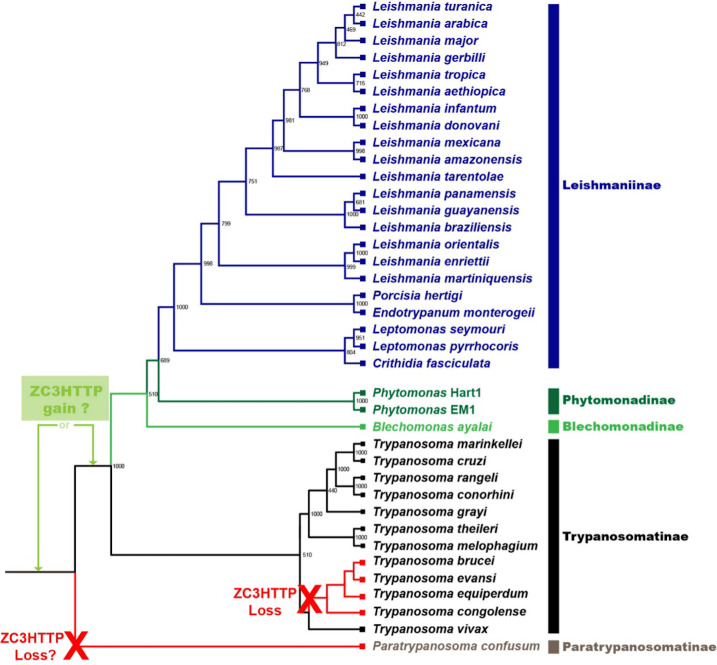
Maximum likelihood tree of trypanosomatids ZC3HTTP proteins. The tree was inferred using the maximum likelihood method based on PhyML analysis for the best evolutionary model. Bootstrap values are shown at the node branches. The tree is drawn to scale, with branch lengths measured in the number of substitutions per site. Colors indicate ZC3HTTP sequences for trypanosomatid subfamily groups.

To understand the TcZC3HTTP role in *T. cruzi* biology, we first investigated when this protein emerged in the *Trypanosoma* family history. Throughout phylogenetic analysis performed with databases from different trypanosomatids combined with protein sequence alignments, we were able to find TcZC3HTTP orthologs in most *Trypanosoma* species, all presenting the zinc finger C3H and DNAJ domains (Fig. 1; Fig. S1; Table S1). Interestingly, this RBP is absent in a branch inside the subfamily Trypanosomatinae that includes the species *Trypanosoma evansi*, *Trypanosoma equiperdum*, *Trypanosoma congolense*, and *Trypanosoma brucei*. Syntenic analysis demonstrated that the tzc3htpp gene correspondent region was not found in these organisms, suggesting that the whole tczc3http gene sequence was lost (Fig. S2). As all the other species, which consist of the entire subfamilies Leishmaniinae, Blechomonadinae, *Phytomonas* sp., and the remaining Trypanosomatinae representatives, along with *T. cruzi*, presented the gene *tczc3http*, it likely appeared in the *Trypanosoma* common ancestor. It is unclear, however, if this RBP was lost on two different occasions or if its appearance occurred only after the Paratrypanosomatinae group speciation and then was lost in the *T. brucei* and related Trypanosomatinae species group. However, it is possible to assume that, besides being ancient, the relationship of TcZC3HTTP with Trypanosomatids seems unique since this protein was found exclusively in this group.

### Impact of TcZC3HTTP on gene expression regulation in *T. cruzi*

In our study, we generated two distinct populations of *T. cruzi* to investigate the role of TcZC3HTTP. The first population, designated as ΔTcZC3HTTP, lacked the TcZC3HTTP gene, while the second population expressed a tagged version of TcZC3HTTP known as TcZC3HTTP-3×FLAG (23). We employed the CRISPR/Cas9 system with two specific guide RNAs (gRNA 99 or gRNA 231) to disrupt the TcZC3HTTP gene in the ΔTcZC3HTTP population, and successful knockout was confirmed by sequencing analysis (23).

**Fig 2:**
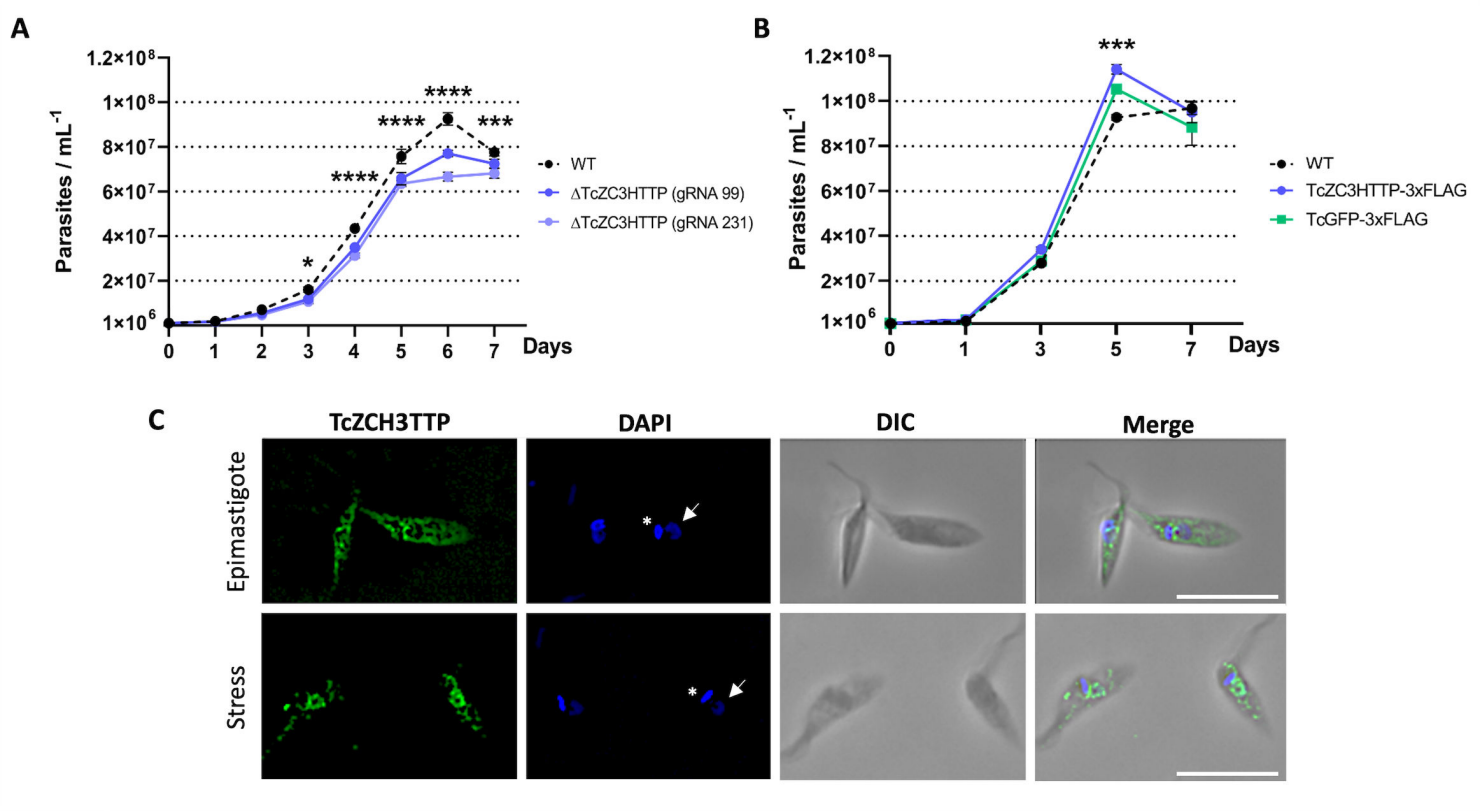
TcZC3HTTP disruption (**A**) or TcZC3HTTP-3×FLAG expression (**B**) impact. Growth curve showing cellular density (*y*-axis) throughout cultivation days (*x*-axis) for null mutants for TcZC3HTTP or parasites expressing TcZC3HTTP-3×FLAG. Wild type (WT) and parasites expressing eGFP-3×FLAG (TcGFP-3×FLAG) were used as controls (*n* = 3; *P < 0.05; ***P < 0.001; ****P < 0.0001). All graphs and statistical analyses were made within the GraphPad Prism software. (**C**) Cellular localization of the TcZF3HTTP-flag protein in epimastigotes under normal growth conditions and parasites subjected to nutritional stress. The commercial monoclonal antibody α-FLAG (1:1,000) was used. The nucleus DNA (white arrow) and kinetoplast (asterisk) were stained with 4',6'-diamino-2-phenylindole (DAPI), and the obtained images were overlaid with differential interference contrast (DIC) (merged). The scale bar represents 10 µm.

Subsequently, we examined the growth characteristics of these populations. The results revealed a significant growth defect in the ΔTcZC3HTTP population compared to the control group, evident from the third day of the growth curve and persisting until the seventh day (Fig. 2A). Conversely, the population expressing the tagged version of TcZC3HTTP (TcZC3HTTP-3×FLAG) exhibited a slightly but statistically significant higher growth rate than the control groups (Fig. 2B). The cellular localization of the protein was also evaluated in epimastigotes under normal growth conditions and in epimastigotes subjected to nutritional stress. It is possible to observe a slight granular pattern in stressed parasites compared to unstressed epimastigotes (Fig. 2C).

These results suggest that TcZC3HTTP expression influences, to some extent, the cell proliferation in *T. cruzi*. The absence of TcZC3HTTP led to decreased growth, while the presence of the tagged protein enhanced cellular proliferation. These observations highlight a relationship between TcZC3HTTP and *T. cruzi*’s cell proliferation and provide valuable insights into its potential functions in the parasite’s life cycle and development.

### TcZC3HTTP RNA targets

**Fig 3:**
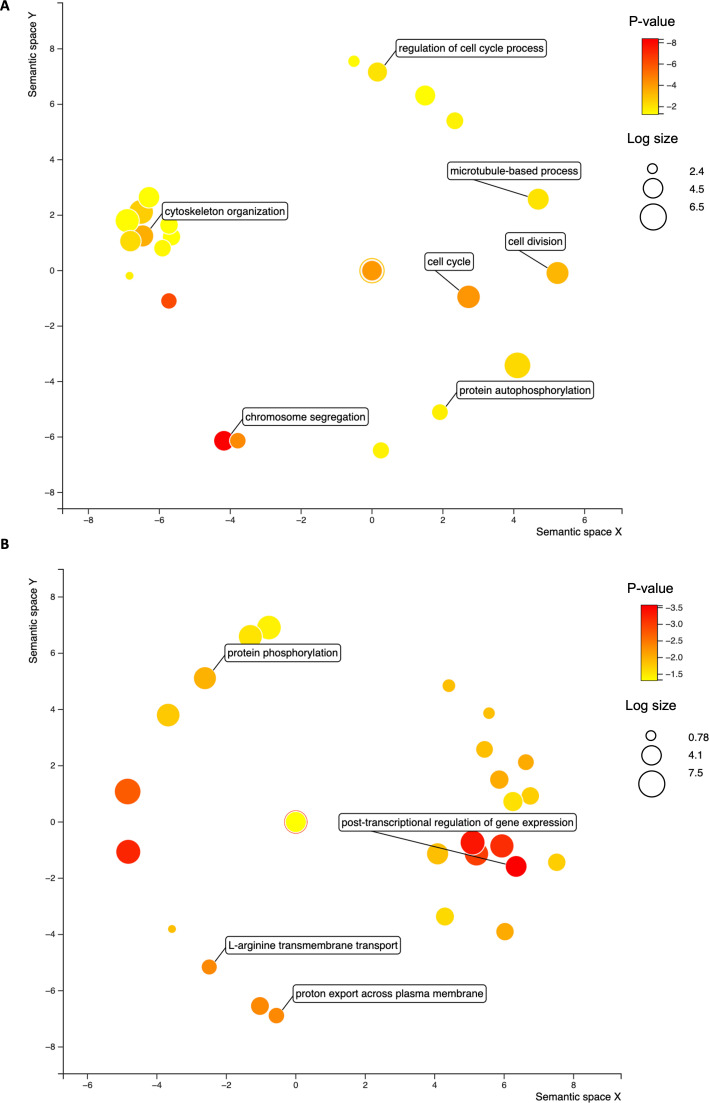
Gene ontology enrichment analysis in the TcZC3HTTP immunoprecipitation. Bubble chart with the gene ontology terms enriched in the RNAs associated with TcZC3HTTP in epimastigotes (**A**) and stressed parasites (**B**). The colors refer to the *P*-value (log-10), and the bubble size is the term count (in log scale). The distance of the bubbles in the *x*- and *y*-axes refers to the proximity of the terms that belong to close-related biological processes.

To elucidate the role of TcZC3HTTP in gene expression regulation in *T. cruzi*, we analyzed the RNAs pulled down with the protein-tagged version of TcZC3HTTP under two conditions: epimastigotes and nutritionally stressed parasites (Fig. 3). Our investigation revealed 243 transcripts associated with TcZC3HTTP-3×FLAG in normal conditions and 159 in stress conditions (Tables S2 and S3). Among these transcripts, 214 were exclusively found in epimastigotes, 130 in stressed parasites, and 29 in both conditions.

Upon querying the Tritryp database for information on these transcripts, we discovered that 40% of the enriched transcripts (148 out of 373) encoded hypothetical proteins. We conducted individual searches for domain predictions and functional indications to gain further insights into these transcripts. The resulting information allowed us to summarize the transcripts that encoded known products associated with the TcZC3HTTP messenger ribonucleoprotein, as depicted in Table 1.

**TABLE 1:** Top transcripts associated with TcZC3HTTP in epimastigotes^*a*^

	TritrypDB ID	Identification	Log₂ FC	FDR *P*-value
RNA metabolism	TcCLB.503453.20	TcZC3HTTP	5.82	0.0%
	TcCLB.503869.40	TcPUF9	2.62	0.0%
	TcCLB.507521.110	TcPUF5	1.86	0.9%
	TcCLB.511815.50	Zinc finger protein family member	2.09	0.2%
	TcCLB.510295.15	ATP-dependent DEAD/H RNA helicase	2.09	2.4%
	TcCLB.506679.140	40S ribosomal protein S10	2.16	0.0%
	TcCLB.503395.50	40S ribosomal protein S12	1.84	0.5%
	TcCLB.511527.34	60S ribosomal protein L8	2.07	0.1%
Cell cycle	TcCLB.511163.40	Cell cycle sequence binding phosphoprotein (RBP45)	3.25	0.1%
	TcCLB.506711.30	Cyclin 3	2.61	5.1%
	TcCLB.511025.120	Cyclin 6	2.51	0.0%
	TcCLB.509507.49	Cyclin 8	1.89	1.3%
	TcCLB.506583.40	Cell division related protein kinase 3 (TcCRK3)	2.52	2.4%
	TcCLB.505183.140	Cell division protein kinase	1.93	0.2%
	TcCLB.508917.10	Polo-like protein kinase (PLK)	2.74	0.0%
	TcCLB.507713.20	WD domain, G-beta repeat	2.86	0.3%
Centrosome	TcCLB.506327.50	Centrosomal protein of 57 kDa	2.77	1.5%
	TcCLB.508919.30	Centrosomal protein of 120 kDa	3.18	0.0%
	TcCLB.508317.20	Centrosomal protein of 162 kDa	3.70	1.0%
	TcCLB.506529.590	Centrosomal protein of 164 kDa	2.59	0.0%
	TcCLB.509717.40	Centrosomal protein POC5	2.06	0.5%
	TcCLB.510101.480	Centriole protein POC11	2.33	0.2%
Kinetochore	TcCLB.507641.190	Kinetoplastid kinetochore protein 1 (KKT1)	2.13	0.1%
	TcCLB.511575.70	Kinetoplastid kinetochore protein 4 (KKT4)	1.87	0.6%
	TcCLB.509429.140	Kinetoplastid kinetochore protein 6 (KKT6)	2.54	0.2%
	TcCLB.506925.490	Kinetoplastid kinetochore protein 7 (KKT7)	2.66	0.0%
	TcCLB.507895.110	Kinetoplastid kinetochore protein 8 (KKT8)	3.59	0.0%
	TcCLB.508543.80	Kinetoplastid kinetochore protein 9 (KKT9)	4.39	0.1%
	TcCLB.509027.60	Kinetoplastid kinetochore protein 10 (KKT10)	1.70	0.5%
	TcCLB.511807.120	Kinetoplastid kinetochore protein 11 (KKT11)	3.32	0.0%
	TcCLB.505071.50	Kinetoplastid kinetochore protein 12 (KKT12)	2.03	2.8%
	TcCLB.507681.250	Kinetoplastid kinetochore protein 14 (KKT14)	2.59	0.1%
	TcCLB.506295.30	Kinetoplastid kinetochore protein 24 (KKT24)	1.91	1.1%
	TcCLB.507221.50	Kinetoplastid kinetochore protein 25 (KKT25)	3.17	0.0%
	TcCLB.419417.19	Kinetochore interacting protein 1 (KKIP1)	4.44	1.8%
	TcCLB.510055.70	Kinetochore interacting protein 5 (KKIP5)	2.71	1.5%
	TcCLB.509353.50	Nucleus and spindle associated protein 2 (NuSAP2)	2.11	0.1%

^*a*^ Experiments were conducted in biological triplicate (*n* = 3).

In epimastigotes, TcZC3HTTP is associated with mRNAs mainly related to kinetochore proteins, cyclins, and centrosomal proteins, and this result reinforces the role of this protein in cell proliferation. We also identified the cell cycle sequence binding phosphoprotein (RBP45) and regulatory elements like the RBPs TcPUF5 and TcPUF9 (Table 1). Accordingly, gene ontology (GO) analysis showed an enrichment of terms involving cell cycle (cyclins), cytoskeleton (centriole), and transcription (Fig. 3A).

Interestingly, we observed that the transcripts with the most significant fold change for stressed parasites were rRNAs (Table S2). Among the mRNAs, the one coding the enzyme phosphoenolpyruvate carboxykinase exhibited remarkable enrichment (fold change > 120) in TcZC3HTTP-3×FLAG immunoprecipitation during nutritional stress. Also, alpha and beta tubulin transcripts were highly represented, with fold changes exceeding 35 and 45, respectively. Furthermore, we discovered that mRNAs encoding the RBPs TcZC3H11, TcZC3H35, TcZC3H28, and TcPUF6 were enriched (Table 2). The significant GO terms associated with this condition were primarily linked to microtubules, ATP binding, zinc finger C3H, and coiled-coil domains (Fig. 3B).

**TABLE 2:** Top transcripts associated with TcZC3HTTP in stressed epimastigotes^*a*^

	TritrypDB ID	Identification	Log₂ FC	FDR *P*-value
RNA metabolism	TcCLB.457295.10	5.8S ribosomal RNA (M3)	8.78	0.0%
	TcCLB.419325.10	Ribosomal RNA small subunit	9.53	0.0%
	TcCLB.473013.10	Ribosomal RNA small subunit	9.48	0.0%
	TcCLB.467341.10	Ribosomal RNA small subunit	8.87	0.0%
	TcCLB.445323.10	Ribosomal RNA small subunit	8.16	0.0%
	TcCLB.447665.10	Ribosomal RNA large subunit alpha	9.01	0.0%
	TcCLB.509005.91	Ribosomal RNA large subunit beta	8.90	0.0%
	TcCLB.411483.20	Ribosomal RNA large subunit gamma (M1)	10.71	0.0%
	TcCLB.422723.20	Ribosomal RNA large subunit delta (M2)	8.46	0.0%
	TcCLB.509005.121	Ribosomal RNA large subunit epsilon (M4)	11.65	0.0%
	TcCLB.508355.260	60S acidic ribosomal protein (P0)	5.29	0.6%
	TcCLB.503395.40	60S ribosomal protein L18	3.76	1.0%
	TcCLB.508823.120	Ribosomal protein S20	5.66	0.3%
	TcCLB.509671.70	Ribosomal protein L15	3.96	4.9%
	TcCLB.506925.120	Eukaryotic initiation factor 5 a (EIF5A)	3.72	5.4%
	TcCLB.510963.90	Elongation factor 2	4.37	0.0%
	TcCLB.508461.140	TcPABP2	4.05	4.6%
	TcCLB.511285.120	ATP-dependent RNA helicase (HEL67)	4.26	3.3%
	TcCLB.503453.20	TcZC3HTTP	2.75	5.1%
	TcCLB.510729.220	TcZC3H28	2.61	2.7%
	TcCLB.511267.24	TcZC3H35	4.17	3.6%
	TcCLB.504929.5	TcZC3H11	3.78	5.1%
	TcCLB.506945.70	MKT1	4.29	3.4%
	TcCLB.510125.10	TcPUF6	3.08	3.4%
Cell cycle	TcCLB.511025.120	Cyclin 6	3.24	2.9%
	TcCLB.511907.260	Cell division cycle protein	4.75	0.0%
Others	TcCLB.510357.10	J-binding protein (JBP1)	2.22	3.9%
	TcCLB.508441.20	Glycosomal phosphoenolpyruvate carboxykinase (PEPCK)	6.95	0.0%
	TcCLB.411235.9	Alpha tubulin	5.15	0.0%
	TcCLB.506563.40	Beta tubulin	5.51	0.0%
	TcCLB.507765.20	PUF nine target 1	4.78	1.9%
	TcCLB.511753.130	U3 small nuclear ribonucloprotein (snRNP)	4.15	3.7%
	TcCLB.507021.20	ESAG8-associated protein	3.89	5.1%
	TcCLB.509247.30	CCR4-NOT transcription complex subunit 1	3.05	0.2%

^*a*^ Experiment conducted in biological triplicate (*n* = 3).

In both experimental conditions, we successfully detected the presence of TcZC3HTTP’s mRNA. Remarkably, we observed a significant enrichment of its transcript in epimastigotes, with a fold change exceeding 50, making it the fourth most abundant RNA identified. Furthermore, TcZC3HTTP mRNA exhibited significant presence even under stress conditions, with a fold change >6. This finding strongly suggests that TcZC3HTTP is involved in an autoregulatory mechanism.

Besides TcZC3HTTP’s transcript, 28 other mRNAs were detected in both experimental conditions (Table S4). Those included transcripts encoding cyclin 6, some kinetochore and cytoskeleton proteins, two kinases, and three nuclear proteins. The remaining mRNAs refer to hypothetical proteins in the database. Notably, 93.1% of these shared RNAs (27/29) presented a higher fold change in nutritionally stressed parasites than in a normal growth context. The only exceptions were TcZC3HTTP’s mRNA and a CMGC/RCK protein kinase.

### TcZC3HTTP protein partners

Given the potential for RBPs to interact with other proteins and the presence of a protein-protein interaction domain (DNAJ domain) in TcZC3HTTP, we used the tagged version of TcZC3HTTP to investigate its candidate protein partners through immunoprecipitation. Following filtering steps, we successfully identified 181 and 55 proteins as modulated under normal and stress conditions, respectively (Tables S5 and S6). Notably, we observed a high enrichment of TcZC3HTTP in our experiment, with fold changes of 133 in epimastigotes and 82.1 in stressed parasites, confirming the efficacy of the immunoprecipitation approach.

Proteomic analysis of the TcZC3HTTP immunoprecipitation revealed the presence of numerous proteins, including hydrogenases, kinases, reductases, peptidases, and tRNA synthetases (Tables 3 and 4). Additionally, we identified chaperones, retrotransposon hot spot proteins, and proteins associated with the proteasome, translation initiation, and elongation factors in epimastigotes. Notably, we observed the association of TcZC3HTTP with several metabolic enzymes, which have been previously recognized as moonlighting proteins (Table 3). This intriguing finding suggests potential functional diversification and the multifaceted roles of TcZC3HTTP in cellular processes.

**TABLE 3:** Proteins associated with TcZ3HTTP-3×FLAG in epimastigotes by immunoprecipitation^*a*^

	TritrypDB ID	Identification	FC	*P*-value
RNA metabolism	TcCLB.503453.20	TcZC3HTTP	133	3.60E−04
	TcCLB.506241.170	40S ribosomal protein S6	7.91	2.20E−02
	TcCLB.511163.40	Cell cycle sequence binding phosphoprotein (RBP45)	11.58	7.00E−03
	TcCLB.510163.20	Elongation factor 1-gamma (EF-1-gamma)	5.71	3.10E−03
	TcCLB.511277.70	Elongation factor Tu, mitochondrial	8.35	7.30E−03
	TcCLB.506925.130	Eukaryotic translation initiation factor 5A	25.48	8.00E−04
Moonlight proteins	TcCLB.511277.290	Aconitase	9.12	2.70E−05
	TcCLB.510945.70	Aspartate aminotransferase	25.54	3.00E−05
	TcCLB.511277.110	Citrate synthase	6.12	2.40E−03
	TcCLB.510943.50	Delta-1-pyrroline-5-carboxylate dehydrogenase	29.09	1.40E−04
	TcCLB.504105.140	Enolase	19.27	2.50E−02
	TcCLB.509287.50	Glucose-6-phosphate 1-dehydrogenase	8.08	1.00E−03
	TcCLB.509717.80	Glyceraldehyde 3-phosphate dehydrogenase, cytosolic	20.63	8.20E−05
	TcCLB.506925.319	Isocitrate dehydrogenase	11.92	3.10E−03
	TcCLB.511419.40	Phosphoglycerate kinase	15.51	5.80E−05
	TcCLB.511281.60	Pyruvate kinase 2	8.5	1.70E−04
	TcCLB.507681.20	Succinyl-CoA ligase	20.74	1.90E−02
Protein folding	TcCLB.506355.50	DnaJ chaperone protein	30.43	4.50E−04
	TcCLB.511465.10	Proteasome activator protein pa26	20.92	3.20E−05
	TcCLB.506985.30	Proteasome alpha 3 subunit	6.92	2.30E−03
	TcCLB.508043.30	Ubiquitin-activating enzyme E1	5.6	2.90E−03
	TcCLB.510661.19	Ubiquitin-activating enzyme E1	6.39	4.40E−03
	TcCLB.506583.30	Ubiquitin-like protein	9.31	1.20E−03
	TcCLB.507029.30	Heat shock 70 kDa protein, mitochondrial precursor	8.01	5.70E−04
	TcCLB.507831.60	Heat shock protein 110	22.22	3.60E−04
	TcCLB.508409.210	Hsc70-interacting protein (Hip)	9.16	9.10E−03

^*a*^ Experiment conducted in biological triplicate (*n* = 3).

**TABLE 4:** Proteins associated with TcZ3HTTP-3×FLAG in stressed epimastigotes by immunoprecipitation assay^*a*^

	TritrypDB ID	Identification	FC	*P*-value
RNA metabolism	TcCLB.503453.20	TcZC3HTTP	82.1	2.80E−04
	TcCLB.508413.50	TcDRBD2	8.06	2.20E−03
	TcCLB.508461.140	TcPABP2	2.37	8.10E−03
	TcCLB.503917.7	TcRBP43	4.9	1.60E−02
	TcCLB.507093.229	TcUBP2	2.17	2.80E−03
	TcCLB.508895.60	TcZC3H40	6.44	3.60E−02
	TcCLB.511285.120	ATP-dependent RNA helicase HEL67	2.36	1.40E−02
	TcCLB.511163.40	Cell cycle sequence binding phosphoprotein (RBP45)	17.44	2.80E−04
	TcCLB.508727.9	CCR4-NOT transcription complex subunit 10	6.89	6.20E−04
	TcCLB.508725.10	CCR4-NOT transcription complex subunit 10	4.65	2.90E−02
Translation	TcCLB.506679.150	40S ribosomal protein S10	4.21	3.60E−02
	TcCLB.508405.40	40S ribosomal protein S11	6.49	3.60E−02
	TcCLB.506297.330	40S ribosomal protein S15A	14.73	8.10E−05
	TcCLB.503899.30	40S ribosomal protein S16	8.16	1.10E−03
	TcCLB.506679.94	40S ribosomal protein S18	6.71	4.60E−02
	TcCLB.506213.60	40S ribosomal protein S2	8.37	1.20E−02
	TcCLB.504021.109	40S ribosomal protein S3	9.13	1.70E−03
	TcCLB.503635.68	40S ribosomal protein S3A	5.08	4.30E−02
	TcCLB.509683.117	40S ribosomal protein S4	8.99	1.00E−02
	TcCLB.506241.170	40S ribosomal protein S6	14.08	2.30E−04
	TcCLB.511903.110	40S ribosomal protein S8	7.22	1.90E−02
	TcCLB.508543.30	40S ribosomal protein S9	8.7	5.40E−04
	TcCLB.503719.20	40S ribosomal protein SA	9.18	7.80E−03
	TcCLB.511277.160	60S ribosomal protein L10a	4.34	1.10E−02
	TcCLB.511727.129	60S ribosomal protein L18a	4.81	4.50E−02
	TcCLB.508461.480	60S ribosomal protein L23	7.52	1.50E−03
	TcCLB.509671.80	60S ribosomal protein L5	8.68	4.20E−04
	TcCLB.504949.14	60S ribosomal protein L6	5.26	6.30E−03
	TcCLB.507519.110	60S ribosomal protein L7	12.17	1.80E−03
	TcCLB.504181.10	60S ribosomal protein L9	10.76	6.90E−03
	TcCLB.503955.70	Eukaryotic translation initiation factor 2 beta subunit	2.29	4.60E−02
	TcCLB.510359.310	Eukaryotic translation initiation factor 2 subunit	2.2	3.30E−02
	TcCLB.510879.120	Ribosomal protein L3	4.29	3.50E−02
	TcCLB.508665.14	Chaperone protein ClpB1, putative	3.93	2.00E−03
	TcCLB.511257.10	Heat shock protein 70 (hsp70)	2.11	2.00E−02

^*a*^ Experiment conducted in biological triplicate (*n* = 3).

Ribosomal subunit 40S and 60S protein enrichment was observed in nutritionally stressed parasites. RBPs were also associated with TcZC3HTTP in this condition, including the zinc finger protein TcZC3H40, as well as the RRMs TcUPB2, TcDRBD2, TcRBP43, and TcPABP2 (Table 4). This result is in accordance with the RNAs associated with TcZC3HTTP in stress conditions. Since TcZC3HTTP was associated with clearly distinct protein groups under normal and nutritional stress, it is reasonable to assume that this RBP can perform different roles according to the cellular context. Furthermore, TcZC3HTTP association with other RBPs and ribosomal proteins during stress, instead of enzymes, implies its participation in the regulatory network rearrangement related to the stress response and is likely associated with a change in translation profiles.

### TcZC3HTTP disruption impacts *T. cruzi* gene expression (RNA)

**Fig 4:**
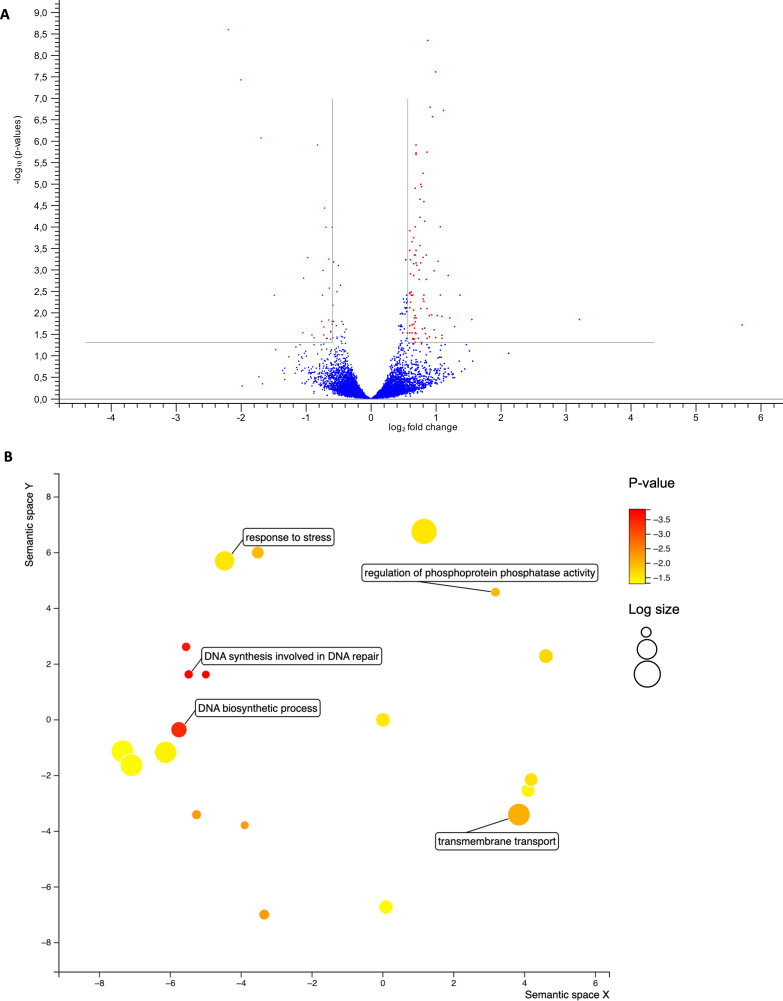
Transcriptomic enrichment analysis in ΔTcZC3HTTP parasites. (**A**) Volcano plot with the mRNAs upregulated or downregulated in parasites lacking TcZC3HTTP against the wild-type population (*n* = 3). Graphs show transcripts differently expressed according to the criteria of the *P*-value (*y*-axis) ≤0.05 and log2 fold change (*x*-axis) ≥ 1.5 or ≤ −1.5 for upregulation or downregulation, respectively. (**B**) Bubble chart with the gene ontology terms enriched in the RNAs upregulated in the DTcZC3HTTP strain compared to the wild type. The colors refer to the *P*-value (log-10), and the bubble size is the term count (in log scale). The distance of the bubbles in the *x*- and *y*-axes refers to the proximity of the terms that belong to closely related biological processes.

One of the primary objectives of this study was to investigate the impact of TcZC3HTTP disruption on epimastigotes and nutritionally stressed cells by analyzing their transcriptome profiles. Our findings revealed differential expression of 126 mRNAs in epimastigotes, with 96 mRNAs upregulated and 30 mRNAs downregulated in the absence of TcZC3HTTP (Fig. 4; Table S7).

In epimastigotes lacking TcZC3HTTP, an increase in the expression of mRNAs encoding proteins related to stress response, transmembrane transport, and DNA synthesis and repair was observed (Fig. 4). The downregulated mRNAs were primarily related to surface proteins and proteins with predicted transmembrane domains. The results also indicated the downregulation of a chaperone DNAJ, several enzymes (including a kinase, alcohol dehydrogenase, metallopeptidase, exonuclease, and a dioxygenase), and the TcZC3HTTP transcript itself (as illustrated in Fig. 4).

Interestingly, the TcZC3HTTP null mutation had minimal impact on the overall mRNA abundance of nutritionally stressed parasites, where only six transcripts were found to be differentially expressed (Table S8). The only upregulated transcript encodes a hypothetical protein with an unknown function domain (DUF229). The downregulated transcripts were an rRNA small subunit, an ubiquitin/ribosomal protein, and another hypothetical protein with no predicted domains. The other two transcripts that were less expressed were related to a protein known as PAD-8, which is associated with differentiation, and were also observed to be downregulated under both normal and stressed conditions (refer to Tables S7 and S8).

**Fig 5:**
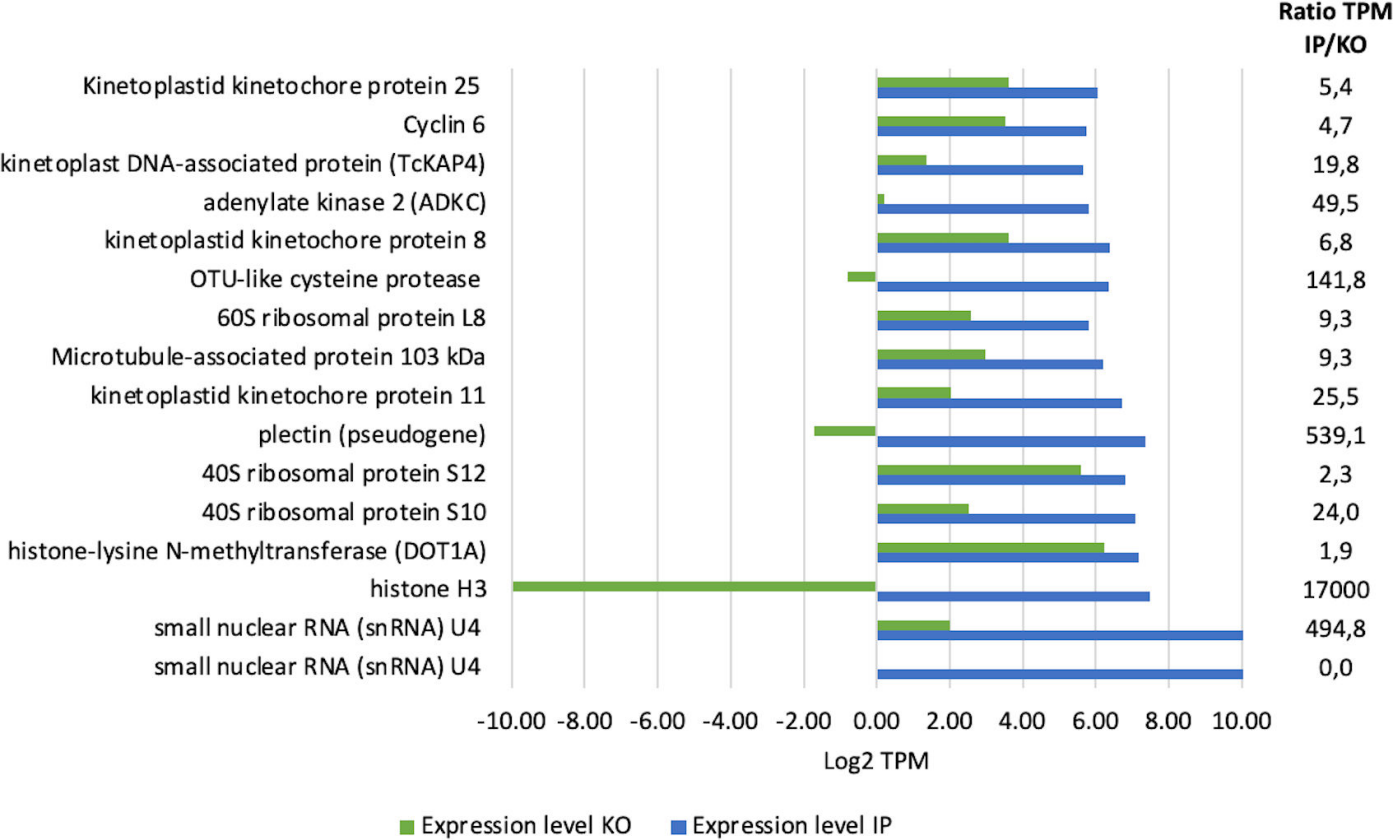
Correlation between mRNAs identified in TcZC3HTTP immunoprecipitation and ΔTcZC3HTTP transcriptomic. Bar chart plotted with the expression values of the transcripts enriched in the TcZC3HTTP immunoprecipitation (IP) assay (green) and the comparison with the expression values in the ΔTcZC3HTTP strain (blue). The *x*-axis represents the expression values in TPM (transcripts per million). The TPM ratio between experiments is shown on the right.

In our investigation, we sought to establish a correlation between the expression values of transcripts associated with TcZC3HTTP in the immunoprecipitation assay and the transcriptome of the knockout strain. Strikingly, the absence of TcZC3HTTP resulted in a noticeable downregulation or reduced expression levels of most of the associated transcripts, implying a potential role for this protein in stabilizing its target transcripts (Fig. 5). This finding underscores the significance of TcZC3HTTP in post-transcriptional regulation and highlights its possible involvement in transcript stability.

### TcZC3HTTP disruption impacts *T. cruzi* gene expression regulation (protein)

**Fig 6:**
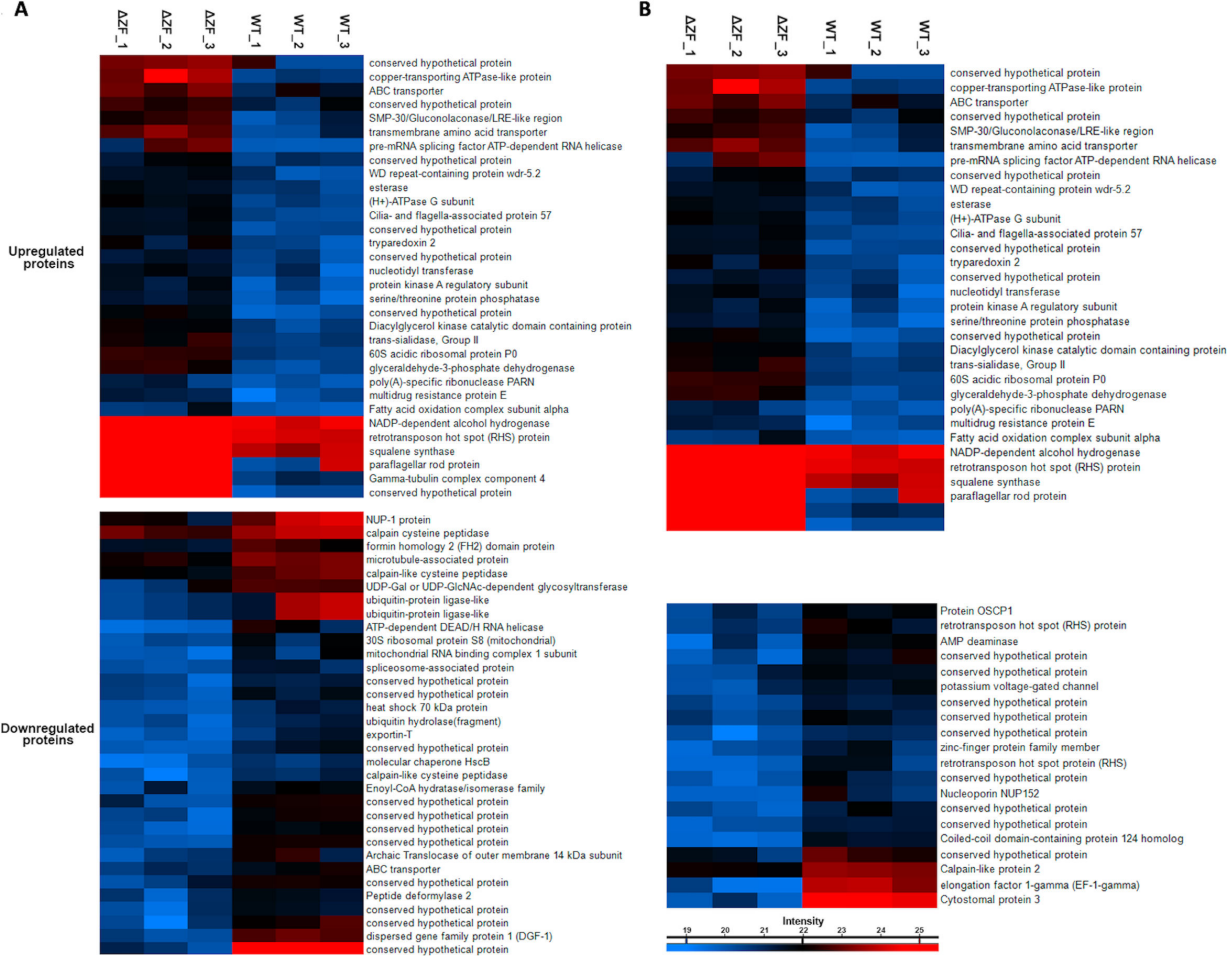
TcZC3HTTP disruption impacts epimastigotes and their nutritionally stressed proteomic profiles. Heat maps showing protein relative expression under normal growth (**A**) or during nutritional stress (**B**) in epimastigotes lacking the TcZC3HTTP gene. In columns ΔZF_1, ΔZF_2, ΔZF_3, and WT_1, WT_2 and WT_3 are respective replicates for the TcZC3HTTP null mutant (ΔTcZC3HTTP) and wild-type populations (*n* = 3). Identified protein expression levels are presented according to the colors from higher (red) to lower (blue) intensities, as indicated in the bars below each heat map set.

We conducted a proteomic analysis under normal and nutritional stress conditions to investigate the impact of TcZC3HTTP absence on *T. cruzi* gene expression. Our results revealed significant alterations in 65 proteins (32 upregulated and 33 downregulated) in epimastigotes, while 59 proteins (39 upregulated and 20 downregulated) exhibited differential expression in nutritionally stressed parasites (Fig. 6; Tables S9 and 10).

In epimastigotes lacking TcZC3HTTP, the upregulated proteins included a component of the gamma-tubulin complex 4, a paraflagellar rod protein, a copper-transporting ATPase-like protein, and a transmembrane amino acid transporter (Fig. 6A). Among the downregulated proteins in normal conditions, we identified two ubiquitin-protein ligases, chaperones, an ATP-dependent DEAD/H RNA helicase, and three calpain-like cysteine peptidases. Furthermore, an exportin and a spliceosome-associated protein were detected (Fig. 6A).

In nutritionally stressed parasites lacking TcZC3HTTP, we observed an upregulation of RBPs, ribosomal proteins, trans-sialidases (group II), and a rad21 double-strand-break repair protein homolog (Fig. 6B). Notably, three proteins exhibited fold changes >10, including the eukaryotic translation initiation factor 3, a conserved hypothetical protein with a predicted rhomboid-like domain, and a protein containing a tetratricopeptide-like helix and a SET domain. Among the 20 downregulated proteins in stressed parasites without TcZC3HTTP, we identified nucleoporin 149, a calpain-like protein, and an AMP deaminase (Fig. 6B). Cystostomal protein 3 and TcEIF1-γ were also downregulated, showing the most significant decreases in expression with fold changes of less than −20 and −15, respectively.

## DISCUSSION

The results present a collection of multiple data points suggesting differential complexes of the TcZC3HTTP with RNA and proteins when parasites are replicating or after nutritional stress. This is confirmed by evaluating the interactions in a parasite lacking the protein. Recently, we employed the CRISPR/Cas9 genome editing system to disrupt TcZC3HTTP and generate null mutants in *T. cruzi* (23). In addition to the TcZC3HTTP null mutant population, we utilized populations expressing a tagged version of TcZC3HTTP to gain further insight into its role in *T. cruzi* gene expression regulation.

As previously described, numerous RBPs have been implicated in and shown to influence critical cellular processes, primarily through the modulation of their target transcripts (11–18). For instance, in *T. brucei*, disruption of the RBP TbRRM1 leads to cell cycle arrest, likely mediated by the modulation of its target TbNOP86, a nucleolar protein crucial for mitotic progression. Downregulation of TbNOP86 results in cell accumulation in the G2/M phase (15). Similarly, in *T. cruzi*, knockout of TcZC3H12 impairs cell proliferation, an effect linked to the targeting and stabilization of mRNAs encoding amino acid transporters (19). When examining the potential target mRNAs of TcZC3HTTP in epimastigotes, we observed an enrichment of transcripts encoding proteins involved in cell growth and division, such as cyclins, centrosomal proteins, and kinetochore proteins. Therefore, it is plausible that the contribution of TcZC3HTTP to cell proliferation is mediated through its target transcript stabilization.

Moonlighting proteins exhibit multiple functional roles, including enzymatic activity and RNA-binding capabilities, showcasing their versatility in cellular processes. Aconitase and glyceraldehyde-3-phosphate dehydrogenase (GAPDH) were both identified as associated with TcZC3HTTP and are prime examples of moonlighting proteins with RNA-binding properties (26–28). Aconitase acts as an enzyme in citrate interconversion and an iron regulatory protein by binding specific transcripts during iron deficiency (29). GAPDH, known for its glycolytic function, also binds RNA, though the underlying mechanism is not fully understood (30, 31). Other metabolic enzymes, such as aldolase, lactate dehydrogenase, and glutamate dehydrogenase, have been implicated in RNA interactions (28, 32). The expanding landscape of RNA-binding proteins encompasses structured RBPs, moonlighting proteins, and unstructured multitasking proteins. Notably, aldolase, trifunctional enzyme subunit b, enolase 1, hydroxymethyltransferase, and pyruvate kinase M2 serve as further instances of RNA-binding metabolic enzymes, illuminating their moonlighting roles in RNA regulation (28, 32, 33). These findings provide valuable insights into the potential interplay between gene expression and intermediary metabolism orchestrated by these versatile RNA-binding metabolic enzymes.

We further identified the RBP TcPUF9 and TcPUF5 transcripts immunoprecipitating with TcZC3HTTP in epimastigotes. TcPUF9 acts to stabilize its targets, and it seems that this occurs specifically in the cell cycle S-phase (11, 34). Although TcPUF9 or TcPUF5 proteins were not identified in the immunoprecipitation assays, we did find one of the TcPUF9 targets (PUF9 target 1) associated with TcZC3HTTP in stressed parasites. RBP45 is a cell cycle-sequence-binding phosphoprotein involved in cell cycle regulation. In *T. brucei*, it is associated with the regulation of transcripts during the S phase. RBP45, TcZC3H39, and TcZC3H40 and their respective orthologs CSBPA and CSBPB interact and form a complex with RBP33 (35, 36). Although RBP33 was not identified in our initial analyses, the mRNA of RBP45 and the protein were co-immunoprecipitated with TcZC3HTTP. This is an important observation, especially considering that TcZC3H40 was detected in the immunoprecipitation of TcZC3HTTP under stress conditions.

During stress conditions, TcZC3HTTP was found to associate with ribosomal RNAs, mRNAs encoding tubulins, kinases, and several other RBPs, including TcZC3H11, TcZC3H35, TcZC3H28, and TcPUF6. It is worth noting that TcPABP2 mRNA exhibited a high enrichment under stress conditions (fold change >15), indicating its potential involvement in stress response, translation profile changes, and rearrangements of RNA granules, where RBPs play a crucial role and coordinate their actions. These findings further support the concept of the cytoskeleton as a regulon, which is consistent with the similarities observed with TcZC3H39 (36).

TcZC3HTTP binds to its mRNA in both studied conditions. This observation has been reported in *T. cruzi* for the zinc finger protein 2 (TcZFP2), which interacts with its mRNA through an A-rich sequence in the 3′ UTR (37). Similarly, tcrbp19 mRNA was identified as a target of its protein, TcRBP19, through its 3′ UTR. However, in this case, TcRBP19 is responsible for negatively regulating its own mRNA rather than stabilizing it (38).

In conclusion, our study generated TcZC3HTTP null mutants in *T. cruzi* and investigated its role in gene expression regulation. We found that TcZC3HTTP potentially modulates target transcripts associated with cell growth and division. Immunoprecipitation assays demonstrated the interaction of TcZC3HTTP with other RBPs, such as TcPUF9, further emphasizing its involvement in RNA regulation. Interestingly, TcZC3HTTP exhibited enrichment with specific mRNAs during stress conditions, suggesting its role in stress response and messenger ribonucleoprotein rearrangements. Overall, our study provides valuable insights into the multifaceted roles of TcZC3HTTP in *T. cruzi* and its contribution to cellular processes and stress responses.

## MATERIALS AND METHODS

### Phylogenetic analysis

The ZC3HTTP sequences from trypanosomatids were obtained by using *T. cruzi* protein (TCDM_03704, ESS67632.1) sequence as a query on BLASTp (39, 40) searches on TritrypDB (41) and GenBank (42) databases. The MAFFT online tool aligned the sequences (43), and poorly aligned positions were discarded by TrimAl (44). The best model with the computation of rates and support was determined by PhyML analysis using the Phylemon 2.0 web suite (45). The consensus phylogenetic tree was visualized and edited using the FigTree v1.4.4 program (available at http://tree.bio.ed.ac.uk/software/figtree).

### *Trypanosoma cruzi* culture and TcZC3HTTP transfected and null mutant parasites

*T. cruzi* Dm28c epimastigotes were cultured at 28°C in liver infusion tryptose medium supplemented with 10% heat-inactivated fetal bovine serum. Cultures overexpressing or null mutants for TcZC3HTTP (TCDM_03704) were obtained as previously described (22). Briefly, 5 × 10^6^ early-log phase wild-type epimastigotes were transfected with 50 µg of pTcGW plasmid (46) containing the gene *tczc3http* (pTcGW-TcZC3HTTP) to generate a TcZC3HTTP-3×FLAG population, selected with G418 (500 µg.mL^-1^) treatment. TcZC3HTTP null mutant parasites were generated from *T. cruzi* Cas9-expressing lineages (for more details, see reference 22) transfected with gRNA targeting the gene tczc3http along with a respective DNA donor to insert a sequence encoding stop codons in three different frames into the target gene. Transfectant and null mutant parasite confirmation are shown elsewhere (23).

### Nutritional stress and cell proliferation analysis

Nutritional stress was performed as previously described (36). Briefly, epimastigotes at the end of the exponential growth phase (5-day cultures at a density of ≥5 × 10^7^ parasites·mL^-1^) were harvested by centrifugation at 5,000 × *g* for 5 min at room temperature, washed twice with phosphate buffered saline (PBS, pH 7.4), and incubated for 2 h at 28°C in TAU medium (190 mM NaCl, 17 mM KCl, 2 mM MgCl_2_, 2 mM CaCl_2_, 8 mM phosphate buffer pH 6.0) at a density of 5 × 10^8^ parasites·mL^-1^. For cell proliferation assay, cultures starting with 1 × 10^6^ epimastigotes·mL^-1^ were monitored throughout 7 days, and parasite density was determined every 24–48 h. We used the automatic particle counter Z Series Coulter Counter (Beckman) to perform the cellular count. All experiments were performed at least in technical and biological triplicates, and one-way analysis of variance statistically analyzed the data. Graphs and statistics were obtained using the GraphPad Prism software version 8.4.2 (La Jolla, CA, USA). The immunofluorescence assay was performed as previously described (23).

### RNA and protein enrichment by immunoprecipitation

To identify potential TcZC3HTTP RNA targets and protein partners, proteins and RNAs were enriched by immunoprecipitation using magnetic beads coupled with anti-FLAG antibodies. Subsequently, 10^9^ parasites expressing TcZC3HTTP-FLAG (in log phase or nutritionally stressed in TAU pH 6.0 for 2 h) were harvested by centrifugation (3,000 × *g* for 5 min), washed with PBS (TAU for stress condition), and lysed accordingly to extract and capture RNAs or proteins. Parasites expressing GFP-FLAG were used as controls and processed in parallel with the TcZC3HTTP-FLAG samples, as described below. For RNAs, parasites were incubated in lysis buffer (NaCl 150 mM, 20 mM Tris-HCl pH 7.4, and Nonidet P40 0.5%) at 4°C for 10 min (lysis was confirmed by optical microscopy). The cellular extract was centrifuged (10,000 × *g* for 20 min at 4°C) and incubated with anti-FLAG M2 magnetic beads (Sigma) for 2 h at 4°C following washing and elution steps using a magnetic separation stand according to the manufacturer’s instructions. RNAs from the eluted fraction were extracted using the miRCURY RNA Isolation Kit Cell & Plant (QIAGEN) and stored at −80°C until cDNA library preparation. For protein isolation and enrichment, cells were incubated with a cold cavitation buffer (20 mM HEPES KOH pH 7.4, 75 mM potassium acetate, 4 mM magnesium acetate, and 2 mM dithiothreitol [DTT]) supplemented with 1× cOmplete Protease Inhibitor Cocktail (Sigma) and 40 µg·mL^-1^ RNase A (Sigma) and disrupted using 1,000 psi (70 bar) for 40 min at 4°C. Next, the lysate was centrifuged (10,000 × *g* for 10 min at 4°C). The supernatant was incubated with magnetic beads Dynabeads M-280 Sheep anti-mouse IgG (Sigma) conjugated with monoclonal ANTI-FLAG M2 antibody (Sigma) for 2 h at 4°C. Later, the magnetic beads were washed, and proteins were eluted in Laemmli sample buffer and stored at −80°C.

### TcZC3HTTP null mutant RNAs and protein extraction

To investigate the impact of TcZC3HTTP absence in *T. cruzi* RNAs and proteins, total RNA and protein were extracted from TcZC3HTTP null mutant epimastigotes in both log phase and nutritionally stressed conditions. As a control, wild-type populations were subjected to the same conditions and processed in parallel with mutants’ samples as follows: total RNA was obtained from 108 parasites that were harvested (3,000 × *g* for 5 min), washed with PBS (TAU for stress conditions), and lysed using the RNeasy Plus micro kit (QIAGEN) following the manufacturer’s instructions. Then, the extracted RNAs were stored at −80°C. For protein extraction, 3 × 10^7^ parasites were used, and after harvesting and washing steps, the cells were lysed in a Laemmli sample buffer and stored at −80°C.

### Transcriptomic analysis

Before proceeding to cDNA library preparation, all RNA samples from three independent experiments (*n* = 3) were quantified, and their integrity was checked. For RNA quantification, a Qubit Fluorometric Quantitation (Thermo Fisher Scientific) RNA BR kit was used. The fragment distribution, integrity, and quality analysis were performed on a 2100 Bioanalyzer (Agilent Technologies, Agilent RNA 6000 Pico Kit). According to the manufacturer’s instructions, the Illumina TruSeq Stranded mRNA kit (Illumina) was used to build the null mutant and IP libraries. The library concentration was measured with the Qubit 4 (Thermo Fisher Scientific) DNA HS assay (Invitrogen), and the 2100 Bioanalyzer DNA 1000 kit (Agilent) was used to determine the medium peak size. Sequencing was performed on a MiSeq (Illumina, v2 pair-end 75 bp run) sequencer at the Carlos Chagas Institute (Fiocruz Paraná). Each sample was individually added to the two lanes as a technical replica to avoid lane-specific errors and improve coverage. Data analysis was performed with CLC Genomics Workbench v20.0.03 software (Qiagen). First, each sample was checked for quality and filtered above PHRED Q30. Reads were mapped against the reference *T. cruzi* genome (CL Brener strain GCA_000209065.1 ASM20906v1) following the parameters: match score (1), mismatch cost (2), linear insertion cost (3), deletion cost (3), length fraction (0.6), similarity fraction (0.8), and global alignment. Differential expression analysis was applied using the mapped read counts. Data were then normalized by the TMM (trimmed mean of M values) normalization method (47). Transcripts were considered differentially expressed if they presented a fold change equal to or superior to 3 in the immunoprecipitation assays and 1.5 in total RNA identification. A false discovery rate (FDR) ≤0.05 was also used. For gene ontology analysis, we used the DAVID functional annotation tool (48). Bubble charts were created using the REVIGO online tool, which uses multidimensional scaling to reduce the dimensionality of a matrix of the GO terms pairwise semantic similarities (49). The guiding principle is that semantically similar GO terms should remain close together in the plot.

### Proteomic analysis

Proteins extracted by immunoprecipitation were analyzed by electrophoresis followed by silver staining and western blot to confirm the presence and enrichment of TcZC3HTTP-3×FLAG. Then, the total protein extracts (approximately 100 µg) and the proteins extracted by immunoprecipitation from three independent replicas (*n* = 3) were separated on 13% polyacrylamide gels. The lanes were reduced with DTT, alkylated, and digested with trypsin. The peptides were extracted with acetonitrile and trifluoroacetic acid, dried, and desalted. Peptides of each sample were separated by online reversed-phase nanoscale capillary liquid chromatography (Ultimate 3000 RSLCnano chromatograph) and analyzed by nano-electrospray mass spectrometry. The mass spectrometry assay was performed at the facility RPT02H of the Carlos Chagas Institute (Fiocruz, Parana) with the Orbitrap Fusion Lumos mass spectrometer. The mass spectrometry data were matched against the *T. cruzi* Dm28c GenBank database (GCA_003177105.1) with MaxQuant software v2.0.3.0 with quantification set to the LFQ method and the following parameters: cysteine carbamidomethylation set to fixed modification, methionine oxidation, and N-terminal acetylation set to variable modification; an FDR of 1% for peptide and protein identification was independently applied. The resulting peptides were analyzed with Perseus v.1.6.14.0. Contaminants, reverse sequences, and peptides only identified by site were removed, the LFQ intensities were transformed to a log2(x) scale, and a normal distribution imputed the missing value. *t*-Tests were performed to determine differentially expressed proteins (*P* ≤ 0.05) and fold change ≥2.

## Data Availability

The RNA-seq data have been deposited at the Sequence Read Archive (SRA) database under the accession number PRJNA986951.

## SUPPLEMENTAL MATERIAL

Supplemental Captions and Figures spectrum.02880-23-s0001.docx Captions for Tables S1 to S12; Figures S1 and S2.

Tables S1 to S10 spectrum.02880-23-s0002.xlsx Additional data for RNA-seq and proteomics.

Tables S11 and S12 spectrum.02880-23-s0003.xlsx Additional data for proteomics.

ASM does not own the copyrights to Supplemental Material that may be linked to, or accessed through, an article. The authors have granted ASM a non-exclusive, world-wide license to publish the Supplemental Material files. Please contact the corresponding author directly for reuse.
