# Supplementary material for: TcZC3HTTP, a regulatory element that contributes to Trypanosoma cruzi cell proliferation
Source: Microbiol Spectr. 2024 Jan 25;12(3):e02880-23. doi: 10.1128/spectrum.02880-23 (PMC10913370; doi:10.1128/spectrum.02880-23)
Supplement: Supplemental Captions and Figures — Captions for Tables S1 to S12; Figures S1 and S2. [file spectrum.02880-23-s0001.docx]

**Supplementary Figures**

**
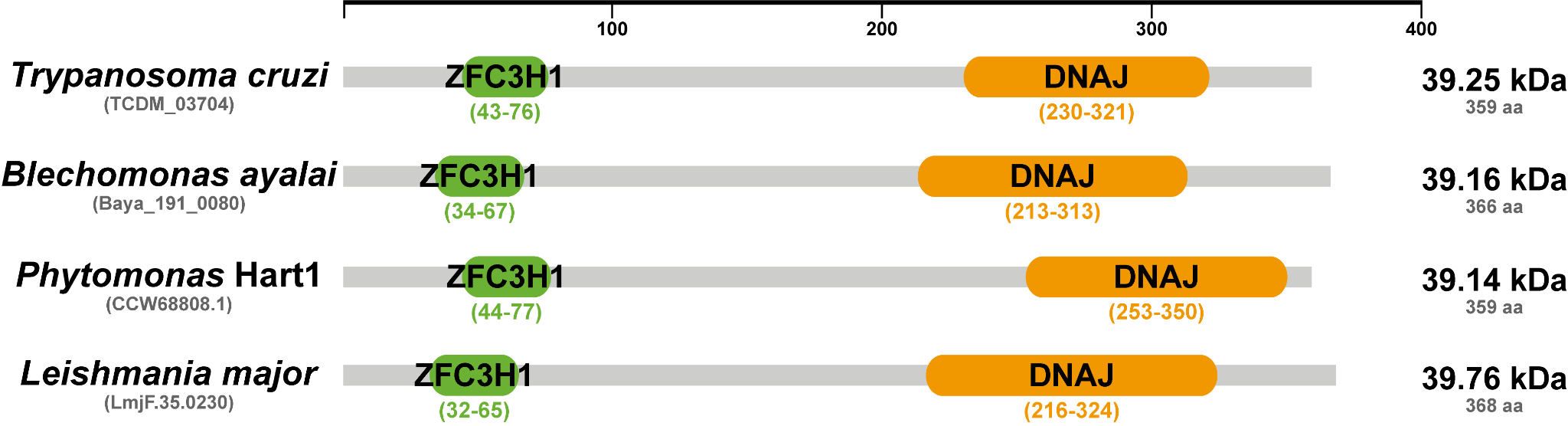
**

**Supplementary Figure 1. Schematic representation of ZC3HTTP homologs from four Trypanosomatids species.** The proteins ZC3HTTP from *T. cruzi, B. ayalai*, Phytomonas Hart1, and *L. major* show similar size and relative positioning for their functional domains, revealing high conservation between groups within trypanosomatids. The full-length proteins are represented in gray, while Zinc finger ZFC3H1 domains are represented in green, and Chaperone DNAJ domains are represented in orange. The domain boundaries were generated through sequence searches using the Interpro online tool (<https://www.ebi.ac.uk/interpro/search/sequence/>)


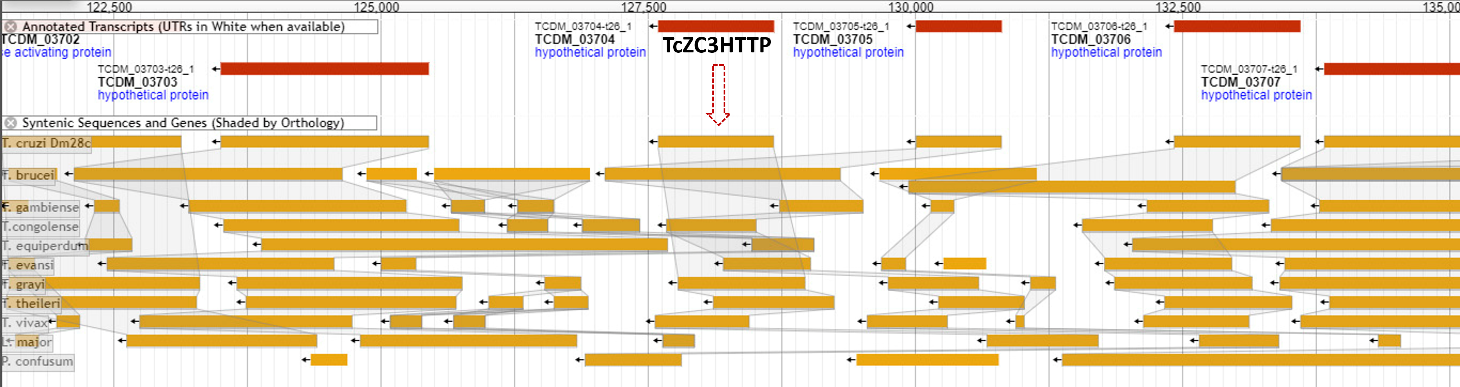


**Supplementary Figure 2. ZC3HTTP gene syntenic analysis across the Trypanosomatids.** The genomic region containing *T. cruzi’s* TcZC3HTTP gene (indicated as TCDM_03704) was compared to other trypanosomatid species (each one depicted in the rows below the dotted arrow). This analysis was performed using the JBrowse online tool (available at tritrypDB.org). Accordingly, tczc3http is present in *T. grayi*, *T. theileri*, *T. vivax*, and *L*. *major*. However, ZC3HTTP’s gene absence in *T. brucei*, *T. congolense*, *T. equiperdum*, *T. evansi*, and *P. confusum*, indicates that in these species a loss of the entire gene occurred. Please note that, due to lack of space, only a few species are represented here, and that this figure is available online. (https://tritrypdb.org/tritrypdb/jbrowse/index.html?loc=AYLP01000029%3A121441..135100&data=%2Ftritrypdb%2Fservice%2Fjbrowse%2Ftracks%2FtcruDm28c2014&tracks=gene%2CSyntenic%20Sequences%20and%20Genes%20(Shaded%20by%20Orthology)&highlight=).

**Supplementary Tables**

Supplementary Table 1. TC3HTTP sequences found in several Trypanosomatid species.

Supplementary Table 2. RNA-seq of the immunoprecipitated transcripts associated with TcZC3HTTP in epimastigote.

Supplementary Table 3. RNA-seq of the immunoprecipitated transcripts associated with TcZC3HTTP in stressed epimastigote.

Supplementary Table 4. Transcripts associated with TcZC3HTTP in normal (EPI) or stress conditions (Stress).

Supplementary Table 5. Proteins associated with TcTZC3HTTP in epimastigote.

Supplementary Table 6. Proteins associated with TcTZC3HTTP under stress conditions.

Supplementary Table 7. Transcriptome of ∆TcTZC3HTTP compared with the wild-type strain in epimastigote.

Supplementary Table 8. RNAs modulated in TcZ3HTTP null mutant nutritionally stressed parasites.

Supplementary Table 9. Proteome of ∆TcTZC3HTTP compared with the wild-type strain in epimastigote.

Supplementary Table 10. Proteome of ∆TcTZC3HTTP compared with the wild-type strain in stressed epimastigote.

Supplementary table S11. Protein groups from the immunoprecipitation analysis (IP-proteomic TcZC3HTTP-3xFLAG x TcGFP-3xFLAG)

Supplementary table S12. Protein groups from the proteomic analysis (ΔTcZC3HTTP x Wild type)
